# Re-assessment of the therapeutic LH window in normogonadotropic IVF patients

**DOI:** 10.3389/fendo.2026.1835446

**Published:** 2026-08-17

**Authors:** Bernadette Mannaerts, Colin M. Howles, Claus Yding Andersen

**Affiliations:** 1 Mannaerts Consultancy, Heesch, Netherlands; 2 ARIES Consulting Sarl, Geneva, Switzerland; 3 Deanery of Biomedical Sciences, University of Edinburgh, Edinburgh, United Kingdom; 4 Institute of Clinical Medicine, Faculty of Health and Medical Sciences, University of Copenhagen, Copenhagen, Denmark; 5 The Fertility Clinic, Copenhagen University Hospital Herlev, Herlev, Denmark

**Keywords:** LH ceiling, LH threshold, LH window, ovarian stimulation, LH/hCG, supplementation

## Abstract

**Introduction:**

Previous research has proposed a luteinizing hormone (LH) threshold and ceiling level, below which oestradiol production is not adequate and above which LH may be detrimental to follicular development. This therapeutic LH window was mainly supported by clinical research in anovulatory patients but observations in normogonadotropic women were controversial.

**Materials and methods:**

A literature narrative review from 1985 through 2025. Studies evaluated included i) preclinical studies using *in vivo* superovulation models in rodents and ii) clinical studies in normogonadotropic IVF patients undergoing OS with recombinant FSH (r-FSH) supplemented with r-LH, urinary human chorionic gonadotropin (u-hCG) or r-hCG or with human menopausal gonadotropin (hMG).

**Results:**

In rodents, small amounts of LH/hCG support FSH-induced multiple follicular development in absence of endogenous gonadotropins, while too high LH/hCG exposure consistently induced follicular atresia. In women treatment with too high doses of GnRH agonist or antagonist resulted in undetectable serum LH (well below 1 IU/L), impaired follicular growth, low rises of serum oestradiol, early miscarriage and reduced chance of pregnancy. Too profound suppression was observed in patients treated with a long GnRH agonist protocol, but the incidence depends on the specific agonist, dose and route of administration. Most women treated with the GnRH antagonist protocol, without other pituitary-suppression, remain within the therapeutic LH window (endogenous LH 1–10 IU/L) during the whole stimulation period. In contrast, too much LH/hCG supplementation can compromise clinical outcome, as confirmed in IVF patients treated with r-FSH and a potent r-hCG in a long GnRH agonist protocol. This r-hCG in daily doses of 1 µg or more inhibited the growth of medium-sized follicles which phenomenon has previously also observed in IVF patients treated with u-hCG and in PCOS patients treated with r-LH during ovulation induction.

**Discussion:**

Whether commercial combination gonadotropins have inhibitory effects on multiple follicular development is difficult to judge and may depend on the protocol, dose of FSH, dose of LH/hCG, number of treatment days and the potency of the specific LH/hCG preparation. It is concluded that normogonadotropic IVF patients undergoing OS have a clearly defined therapeutic LH window below and above which clinical outcome is compromised.

## Introduction

Most women with regular menstrual cycles are normogonadotropic and have serum luteinizing hormone (LH) levels within the normal range. Women undergoing ovarian stimulation (OS) are often pituitary suppressed by gonadotropin-releasing hormone (GnRH) analogues which lowers their endogenous LH. The amount of LH activity needed during OS to provide optimal clinical outcome has already been debated for many years (1, 2).

The question around the need for LH or human chorionic gonadotropin (hCG) supplementation started with the development of r-FSH, which was the first preparation without any LH activity. It was confirmed that women with hypogonadotropic hypogonadism (HH) could develop large pre-ovulatory follicles by treatment with r-FSH alone (3), but proper oestradiol (E_2_) synthesis required some endogenous or exogenous LH activity, in line with the two-cell two gonadotropin concept (4). In most *in vitro* fertilization (IVF) patients a long GnRH agonist protocol using r-FSH for OS was safe and effective, but women with too profound pituitary-suppression were reported to have a compromised clinical outcome. The discussion about LH/hCG supplementation continued after the development of the GnRH antagonist protocol although in this regimen serum LH remains higher during ovarian stimulation. Still today many clinicians provide LH/hCG supplementation during OS as they believe that would improve the chance of pregnancy (5). However, could it be that the amount of LH activity becomes too high during stimulation and reaches the LH ceiling? This overview provides a detailed analysis of LH/hCG levels in women down-regulated with GnRH agonist, in women pituitary-suppressed with GnRH antagonist and evaluates the clinical outcome of women receiving LH/hCG supplementation during OS with r-FSH.

## Methods

This narrative review was conducted through a literature search in PubMed. The following search terms were used: LH threshold, LH ceiling, endogenous LH, exogenous LH, hMG, recombinant LH, urinary hCG, recombinant hCG, LH and hCG supplementation, ovarian stimulation, IVF and/or *in vivo* ovulation models. The literature search included a period of 40 years starting in 1985 (start development r-FSH) up to and including 2025. Studies using *in vivo* superovulation models in rodents and clinical studies in normogonadotropic IVF patients with r-LH, u-hCG, r-hCG and hMG regardless their specific design, were evaluated for their relevance of this review. The therapeutic window for LH was defined by respectively a lower limit and an upper limit of LH activity during ovarian stimulation, below and above the chance of pregnancy is proven compromised. The LH threshold was indicated by the retained endogenous LH following too profound pituitary suppression, whereas the LH ceiling was indicated by too much daily LH/hCG supplementation and related LH/hCG exposure, both resulting in a compromised clinical outcome.

## Results

### *In vivo* rodent models with no or very low gonadotropins

**Figure 1:**
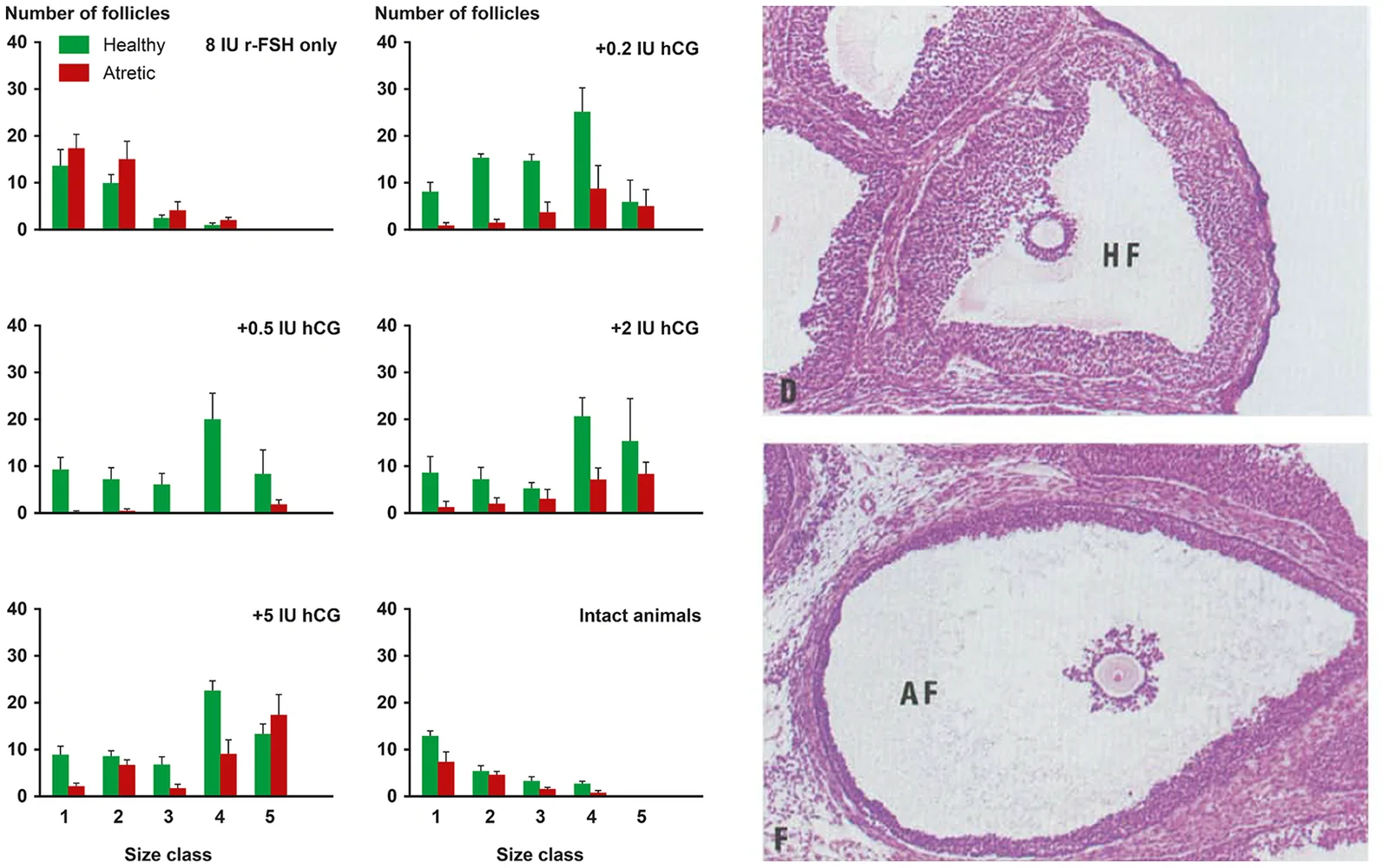
Impact of treatment with r-FSH only or with r-FSH and increasing amounts of hCG on the number of healthy and atretic follicles (left) and ovarian section (right) following treatment with 8 IU r-FSH and 0.2 IU hCG (D) or with 2.0 IU hCG (F). Taken from Mannaerts et al., 1994 (6).

There are few *in vivo* models using rodents with no or very low levels of endogenous gonadotropins examining the role of LH/hCG during OS with FSH alone. Early pharmacology experiments have demonstrated that in hypophysectomised rats, the addition of small amounts of u-hCG (0.2 or 0.5 IU) to a fixed r-FSH dose (8 IU) did not increase the number of follicles but increased the percentage of healthy follicles in comparison to r-FSH alone, whereas the add-on of higher u-hCG amounts (2 or 5 IU) caused follicular atresia (see **Figure 1**). The authors suggested that the number and quality of follicles is determined by the FSH/LH ratio applied for OS and that a favourable ratio is for example 16 rather than 4 (6).

A study in immature mice, showed that both FSH and LH activity are needed to obtain follicular development and maturation of healthy preovulatory follicles containing oocytes capable of undergoing blastocyst development *in vivo* (7). Optimal and maximal follicular stimulation and maturation were achieved with the administration of 1 to 10 IU u-hCG concomitant with 20 IU u-FSH. On either side of this plateau of hCG, follicular development and maturation were severely compromised, resulting in the formation of a significantly lower number of blastocysts. The total hCG activity accounting for as little as 5% of the FSH activity allowed for maximal recovery of pre-embryos, remaining constant while increasing the hCG activity to 50% of the FSH activity; hCG activity outside this range compromises the follicular response drastically. Extrapolating the results from these mice experiments to women indicates women with low circulating levels of LH may benefit from the coadministration of hCG comprising at least 5% but not more than 50% of the FSH dose administered.

The impact of r-FSH (follitropin delta) in combination with r-hCG (choriogonadotropin beta, CG-beta) in a juvenile rat ovulation model was reported at ESHRE in 2023 (8). Recombinant FSH (follitropin delta) induced a bell-shaped dose-response curve for oocyte release with a maximum response of 40–50 oocytes at 8–10 mg/kg r-FSH. The add-on of CG beta potentiated the effects at low to-mid r-FSH doses but had inhibitory effects on the number of ovulated oocytes at high CG beta concentrations. Histology data indicated many cystic follicles following high CG beta exposure which may represent atretic follicles prior to triggering follicular maturation and ovulation. There was no difference between CG beta and CG alfa for the dose effect on the number of ovulated oocytes or ovarian weight. The lowest CG beta dose that clearly reduced the number of ovulated oocytes was 2.4, 0.6 and 0.3 mg/kg in combination with a fixed dose of 1, 3 and 10 mg/kg r-FSH, respectively, which indicated that the rat ovary becomes more sensitive for the inhibitory effect of hCG when higher FSH doses are applied for OS (8).

In summary, *in vivo* rodent models with no or very low endogenous LH suggest that low hCG amounts during OS promote the development of healthy follicles whereas too high hCG exposure induces follicular atresia. The hCG dose causing an inhibitory effect on follicular development may be inversely affected by the FSH dose applied for OS stimulation; thus, the higher the FSH dose the lower the LH ceiling.

### Endogenous LH following long GnRH agonist and GnRH antagonist protocols

**Figure 2:**
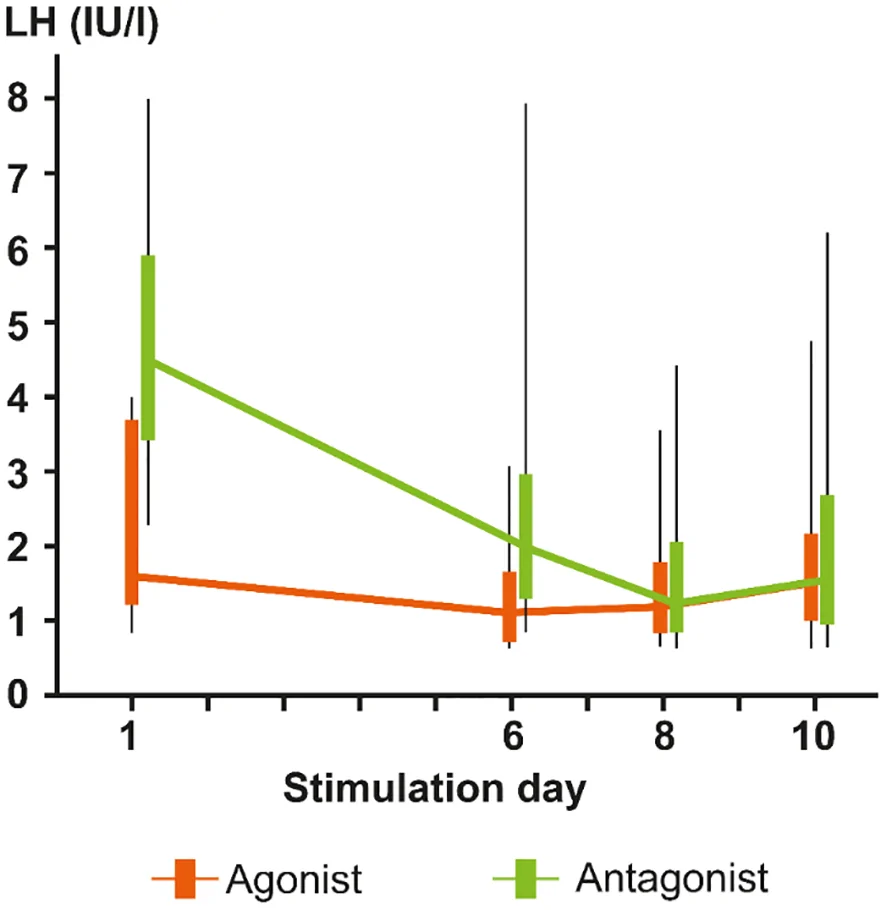
Serum LH concentrations during stimulation with r-FSH for patients with at least 9 days of stimulation in women treated with 0.25 mg ganirelix from day 6 onwards (solid line) and with a long protocol of buserelin (dotted line). The boxes indicate the 75% and 25% percentiles, the vertical lines indicate the 95% and 5% percentiles, and median values are connected. Taken from The European Orgalutran Study Group, Borm and Mannaerts 2000 (9).

Comparative, randomized trials (RTCs) of ganirelix in normogonadotropic women, have specified the retained LH immunoactivity by central laboratory during OS with r-FSH in a GnRH antagonist protocol in comparison to a long GnRH agonist protocol (9–12). In each RCT, a different long GnRH agonist protocol was used in the reference arm. Following agonist down-regulation, endogenous LH was low (median 1 to 2 IU/L) but decreased further to undetectable levels during the first days of stimulation. Some GnRH agonists are more potent than others, thus typically daily 0.1 mg s.c. triptorelin provides more LH suppression at stimulation day 1 than daily 1.0 mg s.c. leuprolide acetate (see **Table 1**; **Figure 2**). Up to 5% of all women treated with daily 0.1 mg triptorelin may have undetectable LH levels (<0.6 IU/L) during the whole stimulation period (see **Table 1**).

**Table 1:** Predose median (5th, 95th percentiles) serum LH levels during ovarian stimulation with r-FSH in three randomized controlled trials (EU study, EU-ME study and NA study) comparing a protocol of daily 0.25 mg ganirelix from day 6 onwards with respectively a long protocol of buserelin (i.n. 0.6 mg/day), of triptorelin (s.c. 0.1 mg/day) and of leuprorelin (s.c. 1.0 mg/day up to down-regulation and 0.5 mg/day thereafter).

	EU study		EU-ME study		NA study	
Day	Ganirelix	Buserelin	Ganirelix	Triptorelin	Ganirelix	Leuprolide
1	4.6 (2.3-7.9)	1.6 (0.8-4.0)	4.5 (2.3- 7.9)	1.3 (<0.6-3.7)	4.8 (2.5-7.9)	3.0 (1.2-6.7)
6	2.1 (0.8-9.2)	1.1 (<0.6-3.1)	1.7 (0.7-7.6)	0.8 (<0.6-2.4)	3.3 (1.0-16.7)	1.8 (0.6-4.2)
hCG	1.6 (<0.6-6.9)	1.5 (<0.6-4.4)	1.5 (<0.6-5.5)	1.0 (<0.6-2.6)	1.7 (0.4-7.6)	1.7 (0.7-4.9)

Taken from references (9–11).

In the investigational arms of the same RCTs, women were treated with ganirelix (0.25 mg/day) starting fixed on stimulation day 6. This implies that serum LH levels were 4 to 5 IU/L at the start of stimulation and decreased during the first days of stimulation due to initial rising serum E_2_ (see **Figure 2**). However, on stimulation day 5 or 6 serum E_2_ may reach a threshold above which it induces the release of LH, also called a premature LH rise if serum LH> 10 IU/L.

The risk of an early LH rise increases with the ovarian response and therefore serum LH levels may largely vary between women during the midfollicular phase. However, high mid-follicular serum LH does not compromise clinical outcome, at least as long as concomitant serum progesterone rises do not occur (13). Following OS with daily r-FSH, the majority of normogonadotropic women had predose LH levels between 1 and 2 IU/L during the late follicular phase and only 5% of the population has undetectable LH levels just before triggering(see **Table 1**; **Figure 2**). Thus the main difference between the two GnRH analogue protocols is with the continuous profound LH suppression during OS in a long GnRH agonist protocol versus the more physiological LH levels during OS in the GnRH antagonist protocol.

In summary, in a long GnRH agonist protocol, serum LH is profoundly suppressed during the whole stimulation period from 1 to 2 IU/L or lower depending on the GnRH agonist, dose and route of administration. In contrast, in a conventional GnRH antagonist protocol serum LH starts with normal levels of 4 to 5 IU/L in the early follicular phase and will decline slowly first due to rising E_2_ and second due to GnRH antagonist treatment to reach 1 to 2 IU/L in the late follicular phase.

### The LH threshold explored in normogonadotropic women treated with GnRH analogues

Following treatment with relatively high doses of GnRH analogues, normogonadotropic women may become hypogonadotropic with undetectable serum LH because of too profound pituitary suppression. Several retrospective analyses have documented that too low endogenous LH during OS with r-FSH may negatively affect clinical outcome. There is evidence that such low LH levels may be induced in IVF patients by the long GnRH agonist protocol as well as by daily high doses with a GnRH antagonist.

One of the first studies on profound LH suppression was from Westergaard (2000) who reported that following a long protocol of 0.5 mg/day s.c. buserelin, 49% of 200 normogonadotropic women had an LH <0.5 IU/L at the day of hCG administration and that these patients had an increased risk of early miscarriage following fresh embryo transfer (14). However, these findings were not confirmed in a retrospective study in 144 women following a long protocol of leuprolide with only 7% of women having LH <0.5 IU/L (15). Using a long protocol of s.c. buserelin, only 12% of 207 women had serum LH levels < 0.5 IU/L and that endogenous LH levels on stimulation day 8 have a positive association with the number of oocytes and an inversed association with the duration of stimulation and the amount of gonadotropins needed for OS (16). In the same year a clinical therapeutic window for LH in controlled OS was suggested with a “threshold” level for LH below which E_2_ production is not adequate and a “ceiling” level for LH above which LH may be detrimental to follicular development. However, no obvious clinical criteria were established to define the group of patients with too profound desensitization (17). Women with HH were used as a model for too profound suppression and in these women supplementation with only 75 IU of r-LH provided more follicles and sufficient E_2_ for endometrial development, whereas supplementation with > 250 IU of r-LH induced atresia of developing follicles both in women with polycystic ovarian syndrome (PCOS) and in women with HH (17). Thereafter, more reports on excessive pituitary-suppression during OS with r-FSH in a long GnRH agonist protocol were published including treatment with 3.75 mg triptorelin depot or daily s.c. 0.1 mg triptorelin (18) and 0.5 mg leuprorelin or 3.75 mg depot triptorelin (19) although the affected outcome parameters varied between studies. Typically, all studies up to then were relatively small and did not allow any conclusions with respect to pregnancy rates.

Larger retrospective analyses were performed to determine the association between endogenous LH and clinical outcome in the conventional GnRH antagonist protocol following OS with corifollitropin alfa (CFA) or daily r-FSH. In these studies, endogenous LH was undetectable (<0.6 IU/L) prior to triggering in 25% of women treated with CFA and in 5% in women treated with daily r-FSH, but ongoing pregnancy rates were not affected by the extent of LH suppression (20, 21),. Similarly, using a LH cut-off value of 0.5 IU/L in a small prospective trial, no differences were noted in clinical outcome of women with undetectable endogenous LH in a GnRH antagonist protocol (22).

**Figure 3:**
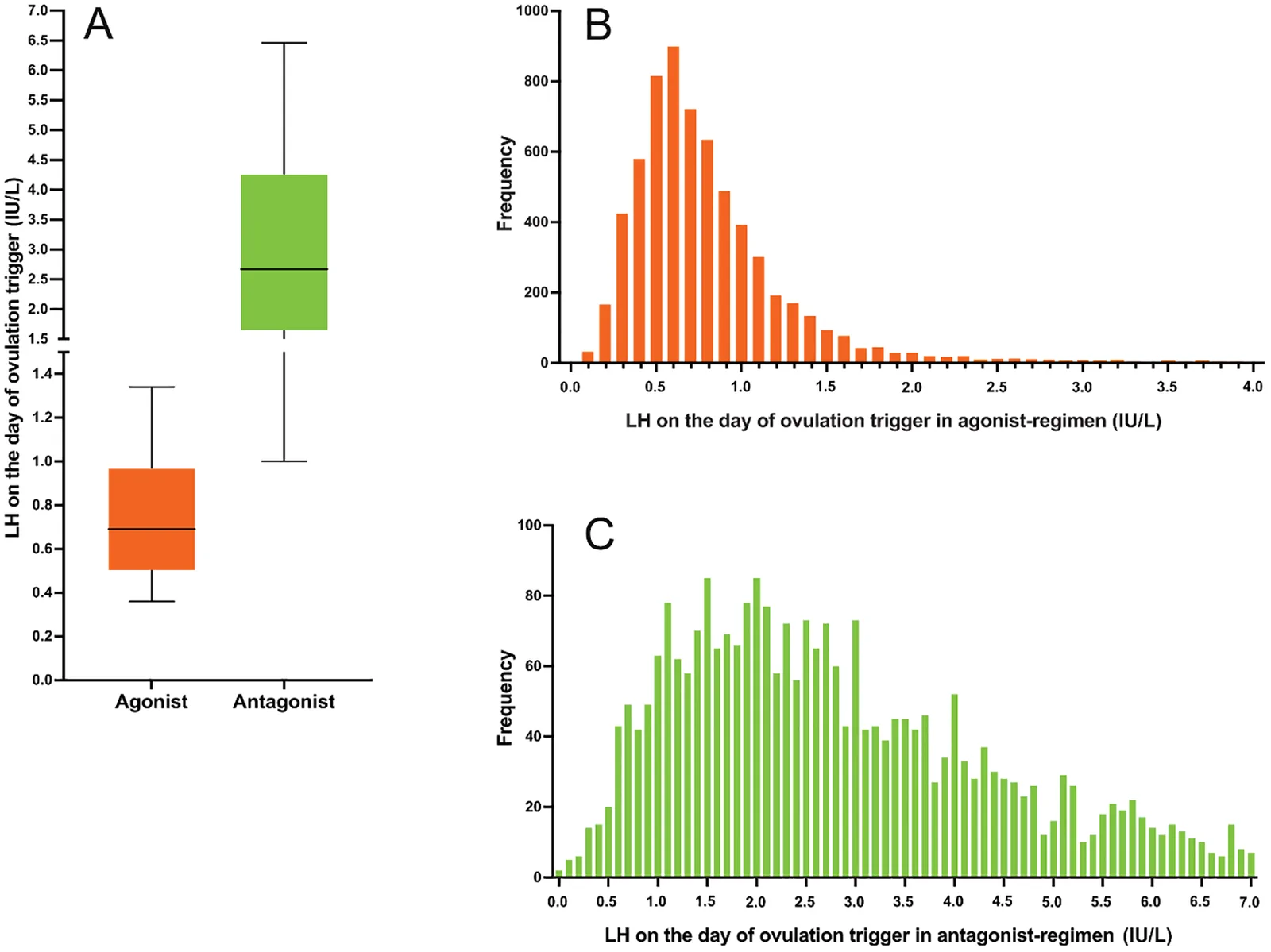
Distribution of serum LH on the day of triggering in long GnRH agonist and GnRH antagonist protocols. **(A)** Box-whisker chart with median, boxes represent interquartile ranges and whiskers represent 10-90^th^ percentiles. **(B, C)** Histograms of serum LH on the day of triggering in long GnRH agonist and GnRH antagonist protocol. Taken from Luo et al., 2023 (23).

However, the best evidence on the impact of too low endogenous LH during OS comes from a very large retrospective analysis in China (23). The authors reported in detail on serum LH levels in a total of 9334 cycles including 6458 cycles in a long GnRH agonist protocol (1.25 mg depot triptorelin) and 2876 cycles in a GnRH antagonist protocol (0.25 mg/day ganirelix or cetrorelix). They compared the distribution of endogenous LH on the day of triggering and reported median (25^th^- 75^th^ percentiles) LH levels of 0.69 (0.5–0.97) IU/L in a long GnRH agonist protocol and 2.67 (1.64–4.27) IU/L in the GnRH antagonist protocol (see **Figure 3**) The GnRH agonist protocol inhibited ~ 90% of patient LH levels to a limit of < 1.34 mIU/mL, while 90% had an LH level > 1 IU/L in the GnRH antagonist protocol. The cycles were assigned into tertile groups based on the LH levels on triggering day which were <0.56; 0.56-0.84; >0.84 IU/L in the GnRH agonist group and <1.98; 1.98-3.61; >3.61 IU/L in the GnRH antagonist group. When comparing clinical pregnancy and live birth rates between the tertiles, the lowest LH tertile had a compromised outcome in the long GnRH agonist protocol but not in the GnRH antagonist protocol.

Too profound LH suppression can also be induced by high dosages of GnRH antagonist as demonstrated in the phase II ganirelix dose-finding study published in 1998 (24). During the development of ganirelix, this prospective, double-blinded, randomized study including 6 different dosages of ganirelix between 0.0625 mg and 2 mg indicated that the effect of increasing ganirelix levels on serum LH levels is immediate and dose-related. The GnRH antagonist was started on stimulation day 6 and following each injection serum LH first decreased to its nadir and increased again to pre-dose levels the next day. The study showed that a low daily GnRH antagonist dose resulted in an increased risk of premature LH rises and a high GnRH antagonist dose resulted in profound LH suppression (day of hCG: 0.4 (<0.25 -0.8) IU/L) and no pregnancies occurred in the 2 mg daily dose group (see **Table 2**). In comparison, the 0.25 mg dose group had LH levels of 1.7 (<0.25-6.4) IU/L and optimal clinical outcome. There were in total 5 women treated with 1 mg or 2 mg antagonist with stagnating follicular growth during antagonist treatment who were switched to hMG treatment. All 5 women finally had embryo transfer, and one became pregnant (24). Because no pregnancies were established in the 2 mg group after fresh embryo transfer, the effect of daily treatment with 2 mg ganirelix on endometrial development was studied in oocyte donors (25). Interestingly, no relevant alteration was observed in the endometrial development in the early and midluteal phases following daily treatment with 2 mg ganirelix in comparison to 0.25 mg ganirelix.

**Table 2:** Median (5^th^ and 95^th^ percentiles) of serum LH concentration on the day of hCG, LH rises and hMG switchers.

Daily dose of ganirelix from stimulation day 6 onwards						
	0.0625 mg N=31	0.125 mg N=65	0.25 mg N=69	0.5 mg N=69	1.0 mg N=65	2.0 mg N=30
LH (IU/L)	3.6 (0.6-19.9)	2.5 (0.6-11.4)	1.7 (<0.25-6.4)	1.0 (0.4-4.7)	0.6 (<0.25-2.2)	0.4 (<0.25-0.8)
LH rise ≥10 IU/L	5	6	1	–	–	–
Switched to hMG	_	_	_	_	1	4

Taken from reference (24).

In summary, an acute hypogonadotropic status may be induced in normogonadotropic women by profound pituitary suppression using a long GnRH agonist protocol or by using high daily doses of GnRH antagonist. A compromised clinical outcome following these scenarios during OS with FSH appears to be associated with very low to undetectable endogenous LH levels (well below 1 IU/L) for several days during ovarian stimulation.

### The LH ceiling explored in women receiving LH/hCG supplementation

The concept of an LH ceiling was suggested for the first time more than 30 years ago and poses that high LH levels suppress granulosa proliferation, and initiates non‐dominant follicle atresia (26). Evidence for the LH effect came first from studies in rat and human granulosa cells, which indicated that high-LH exposure negatively regulates granulosa cell growth while positively regulating steroid synthesis (27, 28). Around the same time several clinical studies showed that in anovulatory women with PCOS too high LH during stimulation may cause premature luteinization which is associated with poor oocyte quality, reduced rate of fertilization, reduced rate of embryo implantation and a high rate of miscarriage (29, 30),. One major challenge specific for ovulation induction of anovulatory PCOS patients is the prevention of multiple follicular development and ovulation. Thus, a multi-center study was undertaken to evaluate if it is possible to minimize follicular development and reduce rates of multiple pregnancy using exogenously administered r-LH (31). In PCOS patients with predefined hypersensitive ovaries, discontinuation of FSH treatment and supplementation with 225 IU or 450 IU r-LH decreased the number of follicles on the day of hCG administration compared to placebo treatment. Therefore, it appears that normal follicular development ceases, leading to atresia, when exogenous r-LH exposure is 225 IU or more per day in the late follicular phase. Confirmation of these data was provided by a dose-finding study in which 153 PCOS patients hyper responding to r-FSH received next to daily 37.5 IU r-FSH, a daily dose of 6.8, 13.6, 30 or 60 µg r-LH (32). Results showed that the proportion of patients developing a single dominant follicle increased up to a daily dose of 30 µg r-LH (660 IU); the number of patients with one leading follicle increased significantly from 13.3% in the placebo group to 32.1% in the 30 µg r-LH group.

That excessive LH/hCG supplementation could be harmful during OS has to date received little attention, probably because PCOS patients are recommended to be treated with r-FSH alone for IVF/ICSI (33) and comparative studies of hMG vs r-FSH suggested that increased hCG levels during OS were positively associated with good-quality embryos and/or implantation rates (34–37). In these studies, hCG supplementation was provided by low daily doses of 20–30 IU hCG (150 to 225 IU hMG) and the ovarian response induced by hMG was lower than for r-FSH. In view of the potential beneficial effect of hCG supplementation, a small, randomized study was designed applying higher daily doses of 50, 100 or 150 IU u-hCG. The study included 62 normogonadotropic women undergoing OS with a fixed daily dose of 150 IU r-FSH in a long GnRH agonist protocol (38). Steady state hCG levels were reached on stimulation day 6 and were prior triggering 3.1 (2.6–3.6) IU/L following daily 50 IU hCG, 5.5 (4.1–7.4) IU/L following daily 100 IU hCG and 11.0 (8.9–13.6) following 150 IU hCG. On the day of triggering, a significant hCG dose-dependent incremental increase was found for progesterone (49–160%), 17-OH-progesterone (223–614%), androstenedione (91–340%) and testosterone (95–338%) from Dose 0 to Dose 150 IU, respectively. This study demonstrated that supplementation with high doses of hCG resulted in a dose-dependent increase in the levels of androgens, progesterone and 17-OH-progesterone confirming that hCG does not lower, but rather increases, preovulatory progesterone levels. Although no statistical difference between the groups was found in terms of the number of follicles per size class or the number of oocytes, the number of 11–14 mm follicles declined in an hCG dose-related manner from 7.9 ( ± 5.8) in the placebo group to 5.3 ( ± 4.4) in the 150 IU hCG group. In addition, the authors reported significantly more day 3 top-quality embryos for patients treated with 150 IU of hCG per day.

**Figure 4:**
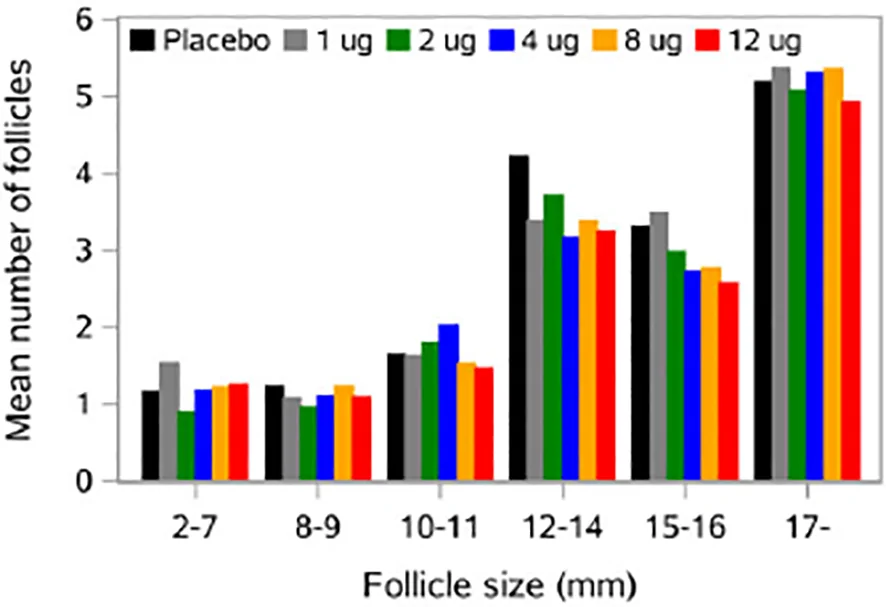
The effect of CG beta on the number of follicles at different size classes at end-of-stimulation. The bars show the mean number of follicles for each size class and treatment. Taken from Fernández Sánchez et al., 2022 (39).

The results of this pilot study encouraged the design of a large Phase II randomized, placebo-controlled study to test a broad range of a new recombinant hCG (CG beta) in a long protocol of triptorelin and the primary endpoint was the number of good-quality embryos (39). Patients (n=100 per group) were randomized to receive either placebo or 1, 2, 4, 8 or 12 µg CG beta added to the daily dose of r-FSH (follitropin delta) during ovarian stimulation. Serum hCG concentrations increased dose proportionally with the dose and steady state concentrations were reached on day 6 of approximately 0.1 ng/mL in the lowest dose group and 1.0 ng/mL in the highest dose group. At the end of stimulation, regardless the CG beta dose, a reduction in the number of intermediate follicles was observed (**Figure 4**) impacting all down-stream parameters, including the number of good-quality blastocysts and pregnancy rates. As in the pilot study with u-hCG, CG beta increased serum E_2_, progesterone, 17-OH-progesterone, androstenedione and testosterone in a dose-dependent manner up to the day of triggering, whereas progesterone declined between triggering and oocyte retrieval, which may indicate LH receptor down-regulation (40, 41).

The outcome of the study confirmed that too much LH/hCG supplementation may inhibit multiple follicular growth by inducing atresia of medium-sized follicles resulting in less oocytes and good quality embryos which compromises the pregnancy chance per treatment cycle. Interestingly, the study tested a broad range of daily 1 to 12 µg CG beta, but a reduction in the number of intermediate follicles was observed in all dose groups, indicating that the LH ceiling was already reached at the lowest test dose in a long GnRH agonist protocol. The lowest dose of CG beta without any inhibitory effect is still unknown and may even be lower in a GnRH antagonist protocol.

### Comparing potency of various gonadotropins containing LH activity

Whether u-hCG in hMG or r-LH in combination gonadotropins have similar effects as CG beta is difficult to conclude and may depend on the dose and duration of administration. The pharmacokinetics (PK) of CG beta has shown to be comparable in men and in women (42). Following a single dose of 125 µg in male volunteers, the mean AUC of CG beta was 1.5-fold greater than of CG alfa, a difference that is mainly caused by the longer elimination half-live of CG beta (47 vs. 32h). In addition, induced serum testosterone reflected the PK profiles with a slight delay, resulting in 59% higher AUC for CG beta (42). Since CG alfa and u-hCG have shown previously a similar PK and pharmacodynamic (PD) profile (43), these findings may be extrapolated to u-hCG.

However, extrapolation of CG beta doses to u-hCG, r-hCG or r-LH applied during OS in IVF patients remains complicated as a direct comparison during OS is lacking. Such comparison is needed as the CG beta doses tested during OS were much lower than in male volunteers and the inhibitory effect, which was relatively small, was not dose-dependent, neither for CG beta (39) nor for u-hCG (38). It has been suggested before that 1 µg CG beta may have a potency similar to 225 IU LH or 30 IU hCG (39), which is present in 225 IU hMG (see **Table 3**), however there is a large insecurity around this estimate and an inhibitory effect by u-hCG may be closer to daily 50 IU as shown previously (38).

**Table 3:** Potency assumptions of CG beta in comparison to commercial combination gonadotropins.

Combination products	FSH bioactivity^a^	LH bioactivity^a^	hCG bioactivity^a^	Compared to CG beta
r-FSH + r-LH (2:1 ratio)	150 IU (11 µg)	75 IU (~3,4 µg r-LH^c^)	10 IU hCG	0.33 µg
	300 IU (22 µg)	150 IU (~6,8 µg r-LH^c^)	20 IU hCG	0.66 µg
hMG: u-FSH + u-hCG (1:1 ratio)	150 IU	150 IU	20 IU hCG	0.66 µg
	225	225 IU	30 IU hCG	1 µg^b^
	300	300 IU	40 IU hCG	1.33 µg
	450	450 IU	60 IU hCG	2 µg

^a^ According Pharmacopeia *in vivo* bioassays in which hCG is about 7-fold more potent than LH (5).

^b^ Estimate taken from Fernández Sánchez et al., 2022 (39), linear extrapolation for other doses

^c^ Specific activity of r-LH (Luveris) has been taken from Hugues et al., 2005 (32)

## Discussion

This review describes the therapeutic LH window in normogonadotropic IVF patients undergoing OS with r-FSH. The concept of the clinical therapeutic window for LH during OS was first described in the 1990’s and the first evidence was mainly based on studies in patients with HH to evaluate the LH threshold and in patients with PCOS to examine the LH ceiling, whereas the evidence in normogonadotropic women was limited and controversial (17). In the current study, the LH threshold is indicated by the retained endogenous LH following too profound pituitary suppression, whereas the LH ceiling is indicated by too much daily LH/hCG supplementation and related LH/hCG exposure, both resulting in a compromised clinical outcome.

In normogonadotropic women, too profound pituitary-suppression may be induced by a long GnRH agonist protocol (23) or by a too high dose of GnRH antagonist (24). The apparent LH threshold may vary between studies as the detection limit of serum LH immunoassays varies between 0.25 and 1 IU/L, which complicates the comparison of results between studies. The LH threshold may also vary between women, but most normogonadotropic IVF patients treated in a long GnRH agonist protocol with serum LH well below 1 IU/L for several days are too profound pituitary-suppressed and may suffer from follicular growth stagnation and impaired estrogen synthesis if treated with r-FSH only. These observations are in good agreement with previous studies in women with HH and illustrate that IVF patients may revert from a normogonadotropic into a hypogonadotropic status. There are no predictors of such a profound suppression following GnRH agonist treatment, but some GnRH agonists are known to be more potent than others mainly due to their high receptor affinity (44). In the past, therapeutic GnRH agonist doses indicated for prostatic cancer were also applied in IVF patients without proper dose-finding studies to assess the minimum effective dose. However, a few studies examined lower GnRH agonist doses and showed that the dose may be reduced without risking premature LH rises, which is often applied in women with a relatively low body weight (45, 46). Overall, there is consensus that patients with too low endogenous LH should receive LH/hCG supplementation by low doses of 75 to 150 IU r-LH or 10 to 20 IU hCG (75 to 150 IU hMG, 47).

In IVF patients treated with the conventional GnRH antagonist protocol using daily 0.25 mg antagonist from stimulation day 5 or 6 onwards, serum LH levels may vary between 1 and 10 IU/L during the early to mid-follicular phase and decrease to average 1 to 2 IU/L in the late follicular phase when 90% of women have an LH level >1 IU/L (23). These LH levels are thought to be within the LH window and suggest no reason for LH/hCG supplementation. The antagonist protocol may also be used in combination with luteal E_2_ pretreatment which has only limited effect on the endogenous LH release (48) and does not affect clinical outcome (49). However, endogenous LH levels may drop below 1 IU/L if the GnRH antagonist protocol is combined with other pituitary suppressive agents like the oral contraceptive pill (50).

On the other hand, IVF patients treated with the conventional GnRH antagonist protocol may also experience too high endogenous LH if an early or late LH rise (≥ 10.0 IU/L) occurs. Especially women with a late LH rise have a lower ovarian response and a lower chance of pregnancy due to premature luteinization (13). In general, the incidence of late LH rises is low and often reflects insufficient antagonist exposure for example in obese women (51) and in women with non-compliant daily antagonist administration (24).

During ovarian stimulation, the impact of an LH rise during the mid- to late follicular phase causing premature luteinization is to be distinguished from daily LH/hCG supplementation that primarily drives androgen production. Since androgen receptor levels in granulosa cells decline during pre-ovulatory follicular maturation, the smaller follicles are more prone to atresia than large pre-ovulatory follicles (52, 53),. Androgen synthesis may also be accelerated by the relatively high daily FSH doses during OS as FSH increases the LH receptor expression and thus the LH-induced response (54) Therefore, too much LH/hCG supplementation during OS prior to IVF or ICSI may inhibit multiple follicular growth by inducing atresia of intermediate follicles.

To date, the potential impact of excessive LH/hCG supplementation has hardly received attention, probably as first data were retrieved in anovulatory patients with HH and PCOS undergoing ovulation induction (31, 32),. In IVF practice, LH/hCG supplementation is routinely applied by low doses of 75 to 150 IU r-LH or by 10 to 20 IU hCG which is thought to improve clinical outcome especially in older patients and/or poor responders (55). However, well-designed RCTs were unable to demonstrate any clinical benefit of r-LH supplementation (56, 57). Following comparative studies of hMG versus r-FSH, it was suggested that hCG supplementation could improve embryo quality, thus the potential beneficial effects of hCG were analysed in a small pilot study with high doses of 50, 100 and 150 IU u-hCG with the number of top quality day 3 embryos as the primary endpoint. The inhibitory effect of hCG was not noticed as the number of women per treatment group (15 to 16) were too small and differences were not statistically significant (37). However, based on a Poisson regression analysis, the authors reported that supplementation with 100 IU or 150 IU hCG from the first day of stimulation did increase the number of top-quality embryos per patient. Accordingly, a large phase II dose-range study of CG beta with the primary endpoint the number of good quality embryos included 100 normogonadotropic women per dose group. However, all test doses from 1 to 12 µg inhibited multiple follicular development significantly by reducing the number of intermediate follicles and no improvement of the blastocyst quality was observed (39). Altogether, the impact of too much LH/hCG supplementation has been shown in normogonadotropic women following treatment with CG beta at a daily dose ≥ 1 µg (39) and in a small pilot study of u-hCG at a daily dose ≥50 IU hCG (38), resulting in serum steady state levels ≥ 0.1 ng/ml CG beta and ≥3 IU/L u-hCG, respectively. The impact of these relatively high hCG doses on multiple follicular development are in good agreement with the impact of equipotent doses of r-LH in HH and PCOS patients (31, 32),. Thus, the endocrine status of the women receiving the LH/hCG supplementation maybe less relevant as it is overruled by the pharmacological action of daily administered exogenous LH activity.

In conclusion, normogonadotropic IVF patients have a clearly defined therapeutic LH window below and above the chances of pregnancy may be compromised. In clinical practice too low endogenous LH may occur following treatment with too high doses of GnRH agonist or with GnRH antagonist in combination with other pituitary-suppressive drugs and may be normalized by a low dose of LH/hCG supplementation. Excessive LH activity during OS may inhibit multiple follicular growth leading to less oocytes per stimulation cycle. Whether the LH ceiling is surpassed may depend on the stimulation protocol as well as the specific compound and dose applied for LH/hCG supplementation.
